# ^18^O-Tracer Metabolomics Reveals Protein Turnover and CDP-Choline Cycle Activity in Differentiating 3T3-L1 Pre-Adipocytes

**DOI:** 10.1371/journal.pone.0157118

**Published:** 2016-06-08

**Authors:** Jay S. Kirkwood, Cristobal L. Miranda, Gerd Bobe, Claudia S. Maier, Jan F. Stevens

**Affiliations:** 1 Linus Pauling Institute, Oregon State University, Corvallis, United States of America; 2 Department of Pharmaceutical Sciences, Oregon State University, Corvallis, United States of America; 3 Department of Animal and Rangeland Sciences, Oregon State University, Corvallis, United States of America; 4 Department of Chemistry, Oregon State University, Corvallis, United States of America; Virgen Macarena University Hospital, School of Medicine, University of Seville, SPAIN

## Abstract

The differentiation of precursor cells into mature adipocytes (adipogenesis) has been an area of increased focus, spurred by a rise in obesity rates. Though our understanding of adipogenesis and its regulation at the cellular level is growing, many questions remain, especially regarding the regulation of the metabolome. The 3T3-L1 cell line is the most well characterized cellular model of adipogenesis. Using a time course metabolomics approach, we show that the 3T3-L1 preadipocyte metabolome is greatly altered during the first 48 hours of differentiation, where cells go through about two rounds of cell division, a process known as mitotic clonal expansion. Short-chain peptides were among several small molecules that were increased during mitotic clonal expansion. Additional indicators of protein turnover were also increased, including bilirubin, a degradation product of heme-containing proteins, and 3-methylhistidine, a post-translationally modified amino acid that is not reutilized for protein synthesis. To study the origin of the peptides, we treated differentiating preadipocytes with ^18^O labeled water and found that ^18^O was incorporated into the short chain peptides, confirming them, at least in part, as products of hydrolysis. Inhibitors of the proteasome or matrix metalloproteinases affected the peptide levels during differentiation, but inhibitors of autophagy or peptidases did not. ^18^O was also incorporated into several choline metabolites including cytidine 5'-diphosphocholine (CDP-choline), glycerophosphocholine, and several phosphatidylcholine species, indicative of phosphatidylcholine synthesis/degradation and of flux through the CDP-choline cycle, a hallmark of proliferating cells. ^18^O-Tracer metabolomics further showed metabolic labeling of glutamate, suggestive of glutaminolysis, also characteristic of proliferating cells. Together, these results highlight the utility of ^18^O isotope labeling in combination with metabolomics to uncover changes in cellular metabolism that are not detectable by time-resolved metabolomics.

## Introduction

Adipose tissue plays a major role in whole-body energy homeostasis. During times of energy excess, white adipose tissue serves as a storage depot, harboring intracellular droplets of lipids. When energy is in short supply, the stored lipids are liberated for utilization by peripheral tissues. Adipose tissue mass is primarily determined by lipid droplet size and adipocyte number. Adipocytes increase in size when energy stores are increasing through expansion, and adipocytes increase in number through the differentiation of precursor cells, a process known as adipogenesis. Adipogenesis occurs primarily during childhood and adolescence [1]. However, new adipocytes are formed during the entire lifespan to replace dying adipocytes or to increase the storage capacity of adipose tissue. The majority of white adipocytes are thought to be derived from tissue-resident mesenchymal progenitor cells. Additionally, bone marrow-derived progenitor cells can accumulate in adipose tissue and differentiate into adipocytes in adults [2, 3].

Adipogenesis can be broken down into early and later phases. The later phase is characterized by lipid droplet formation and triglyceride accumulation. In the early stage, a transcription cascade is initiated, where CCAAT/enhancer-binding protein (C/EBP) β activates the expression of peroxisome proliferator-activated receptor γ (PPARγ) and C/EBPα, both considered master regulators of adipogenesis [4, 5]. Also early in adipogenesis, preadipocytes undergo about two rounds of cell division, a process known as mitotic clonal expansion [5].

Mitotic clonal expansion (MCE) occurs within the first 48 hours of adipogenesis and is required for complete differentiation of cells in culture [4,5]. Recent evidence suggests MCE may also play a significant role during adipogenesis in animals, perhaps extending to humans. Merkestein et al. [6] recently discovered that the fat mass and obesity-associated (FTO) gene influences adipogenesis by regulating MCE. The FTO gene was the first gene with common variants that affect susceptibility to obesity in the general population [7].

Many natural products that inhibit adipogenesis in cell culture have been shown to act by affecting MCE. Rohitukine [8], hirsutenone [9], piceatannol [10], and dehydrodiconiferyl alcohol [11] are few among many natural products that have recently been found to inhibit adipogenesis by interfering with MCE. In view of recent findings that adipogenesis also occurs in adults, small-molecule inhibitors of adipogenesis that affect MCE may find clinical application in the treatment of obesity.

The transcriptional, translational, and post-translational events that occur during adipogenesis have been under fairly intense scrutiny [12–14]. Studies focusing on the impact of endogenous metabolites have identified several modulators of adipogenesis, including polyamines, branched-chain and other amino acids, and cellular anti-oxidants. Polyamine levels increase during adipogenesis, and their depletion inhibits adipogenesis [15–17]. It was recently found that polyamines are required for efficient activation of both C/EBP β and MCE [17].

Amino acid metabolism is also involved in adipogenesis. Green et al. [18] found that catabolism of branched-chain amino acids contributes to both adipogenesis and lipid accumulation. Using isotopically labeled metabolic tracers, the authors determined that protein degradation was largely supporting the increase in branched-chain amino acids that were used for lipid synthesis. The specific protein degradation processes or enzymes responsible for the production of branched-chain amino acids and other lipogenic products are largely unknown. It is known, however, that several proteolytic pathways are active during adipogenesis, including autophagy [19], proteasomal degradation [20], and matrix metalloproteinase-mediated proteolysis [21].

Reactive oxygen species (ROS) also play a significant role during adipogenesis. Glutathione, a tripeptide antioxidant present intracellularly at millimolar concentrations, exists in oxidized (GSSG) and reduced (GSH) forms. During adipogenesis, the ratio of GSSG to GSH increases. Antioxidants that prevent the oxidative conversion of GSH into GSSG inhibit adipogenesis, while inhibitors of GSH synthesis stimulate adipogenesis [22]. ROS activates adipogenesis in part through regulation of MCE [23].

In this study, we used a metabolomics approach to uncover changes in small molecules during adipogenesis using the common model 3T3-L1 cells. We specifically focused on the first 48 hours, i.e., during MCE, because metabolic alterations associated with MCE may reveal novel endpoints and targets for developing anti-obesity therapies. Our results regarding changes in polyamines, amino acids, and glutathione agree well with the literature on adipogenesis [16, 22, 24–26]. In addition, we found an increase in several short-chain peptides of unknown origin. The levels of the peptides were altered by an inhibitor of the proteasome (epoxomicin) or a broad spectrum metalloproteinase inhibitor (batimastat). Using cells exposed to isotopically labeled water (H_2_^18^O), we determined that the peptides are products of proteolysis. These labeling experiments also revealed glutaminolysis and CDP-choline cycle activity in differentiating 3T3-L1 cells. Taken together, our isotope-assisted metabolomics approach highlights the metabolic alterations during MCE and provides a set of metabolic endpoints for studies aimed at developing inhibitors of MCE.

## Materials and Methods

### Chemicals

Bafilomycin A1, epoxomicin, bestatin, and EUK134 (chloro[[2,2'-[1,2-ethanediylbis[(nitrilo-κN)methylidyne]]bis[6-methoxyphenolato-κO]]]-manganese) were from Cayman Chemical Company (Ann Arbor, MI, USA). MnTMPyP (Mn(III)tetrakis(1-methyl-4-pyridyl)porphyrin) was from ENZO life sciences (Farmingdale, NY, USA). 3-Isobutyl-1-methylxanthine (IBMX), dexamethasone (DEX), insulin, hydrogen peroxide, epoxomicin, batimastat, and H_2_^18^O were from Sigma (St. Louis, MO, USA). Chemical standards used for metabolite identification were from TCI America (Portland, OR, USA) and when unavailable, Sigma.

### Cell culture and treatment

The 3T3-L1 cell line used here was from ATCC (Manassas, VA, USA). Cells were first propagated in 75 cm^2^ flasks using a culture medium consisting of DMEM (Life Technologies, Grand Island, NY, USA), 10% fetal bovine serum, 100 units/ml penicillin, 100 μg/ml streptomycin, and 1 mM pyruvate. Differentiation of 2-day post confluent (designated day 0) 3T3-L1 cells was induced by a standard chemical cocktail, designated MDI, which included insulin (200 nM), dexamethasone (250 nM), and 3-isobutyl-1-methylxanthine (500 μM). When cells were differentiating in the presence of metalloporphyrin complexes, MnTMPyP and EUK134 were added 30 min prior to the MDI cocktail at a final concentration of 40 μM. PEG-catalase was incubated with cells 18 h prior to MDI addition. Hydrogen peroxide (H_2_O_2_) was added 30 min after MDI and cells extracted 12 h later. Epoxomicin, bafilomycin A1, bestatin, batimastat, and H_2_^18^O were added 12 h after MDI. H_2_^18^O or control H_2_O was added at a final level of 6% by volume.

### LC-MS/MS based metabolomics

Metabolomics experiments, including metabolite extraction, data collection, statistical analysis, and metabolite identification, were performed as previously described [27, 28]. Briefly, metabolites were extracted with cold solvent (50:50 MeOH:EtOH v:v), centrifuged, then the supernatant was analyzed. MeOH:EtOH was chosen as the extraction solvent because it efficiently precipitates proteins yielding a clean extract, and it extracts both polar and nonpolar metabolites. Metabolites were separated on an Inertsil Phenyl-3 stationary phase (GL Sciences) coupled to a quadrupole-time-of-flight mass spectrometer (Triple TOF 5600, AB SCIEX) with MS/MS spectra recorded on the fly on an information-dependent basis. Only spectral features with peak areas greater than 10,000 units were included for further analysis. Metabolites were identified using an in-house library of standards based on accurate mass (error tolerance < 10 ppm), fragmentation pattern (library score > 70), isotope distribution (difference < 10%), and retention time (error tolerance < 9%). A list of identified metabolites can be found in S1 Table.

### Determination of cellular protein content

The change in total protein content of differentiating adipocytes was determined using the Coomassie Plus Protein Assay Reagent (Pierce, Rockford, IL) according to the manufacturer’s instructions. At 24 h post differentiation, cells were washed once with HBSS, then 500 μL cold lysis buffer was added to each well of the 6-well plate. Lysates or bovine serum albumin (to generate a standard curve) was diluted with water into 96-well plates, then Coomassie Plus reagent was added and after a 10-min incubation, the absorbance was read at 590 nm using a SpectraMax 190 plate reader.

### OCR and ECAR measurements

Oxygen consumption rate (OCR) and extracellular acidification rate (ECAR) measurements were performed with a Seahorse XF 24 Analyzer from Seahorse Bioscience (North Billerica, MA, USA). 3T3-L1 Fibroblasts were plated at a density of 30,000 cells per well and then differentiated as described above 2 days after reaching confluence. XF medium from Seahorse Bioscience was used for OCR and ECAR measurements and contained 25 mM glucose, 1 mM pyruvate, and 200 nM insulin.

### Statistical analysis

Statistical analyses were performed using GraphPad Prism 4.0 software and SAS 9.3 software. In studies with only two experimental groups, we compared the two groups using Student’s unpaired t-test. If there were more than two groups, we used an ANOVA and compared the groups using Tukey’s range test, which is a post hoc test that accounts for multiple comparisons. All statistical tests were two-sided. Significance was declared at p < 0.05; the exception is the untargeted metabolomics study, in which significance was declared at q < 0.05, using the adaptive linear step-up procedure of Benjamini [29] to control for the false discovery rate.

## Results and Discussion

### Time course metabolomics reveals significant alterations in the 3T3-L1 preadipocyte metabolome shortly after initiating differentiation

**Fig 1:**
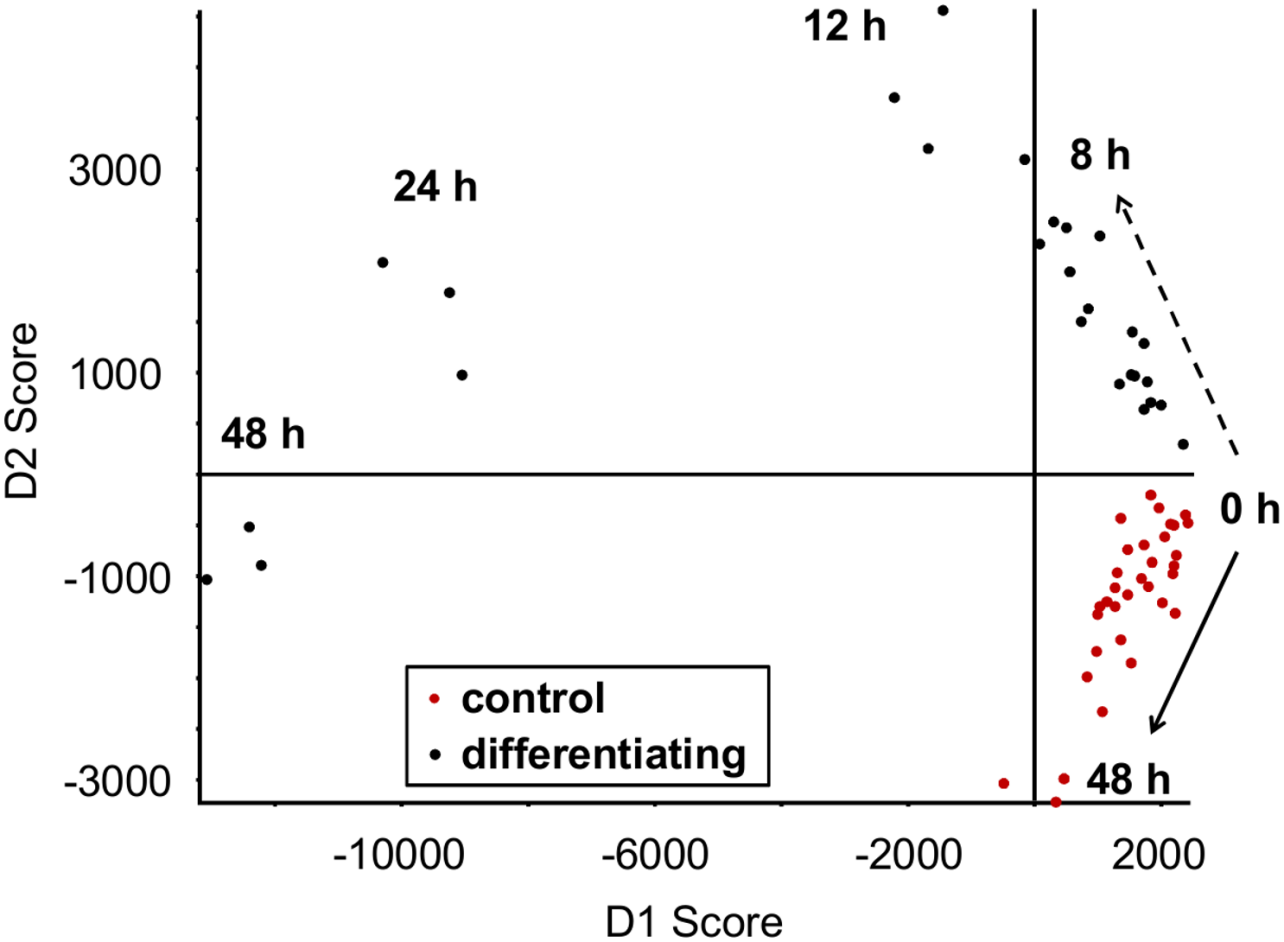
Time course metabolomics of differentiating adipocytes. PCA-DA scores plot representing metabolomic analysis (positive ion) of differentiating and control (0.1% DMSO) 3T3-L1 fibroblasts during the early phase of differentiation. The times refer to the amount of time passed after the addition of the differentiation cocktail or DMSO. n = 3 biological replicates for all time points except 0 h, where n = 6. Shown are means ± SE.

To determine the effect of differentiation on the adipocyte metabolome, 3T3-L1 preadipocytes were extracted at several time points (10 min, 1 h, 2 h, 3 h, 4 h, 6 h, 8h, 12 h, 24 h, and 48 h) after chemically initiating differentiation into mature adipocytes. In addition to potentially uncovering novel differentiation-associated changes in cellular metabolism, this method allows us to track over time the metabolite changes that have been reported previously in the early stages of adipocyte differentiation, validating this method and providing temporal data for several differentiation-associated metabolic switches. A principal components discriminant analysis (PCA-DA) plot shows tight sample grouping and clear group separation of the adipocytes extracted 8–48 h post differentiation (Fig 1) suggesting significant alterations to the metabolome early in the differentiation process. The greatest changes were observed at the 24-hour time point, which we examined in more detail. A total of 16,922 spectral features were detected 24 hours post differentiation. To adjust for multiple comparisons, q-values were calculated using the adaptive linear step-up procedure of Benjamini [29] that controls the false discovery rate. All statistical tests were two sided and considered significant at q < 0.05. From the detected 16,922 features, 77 metabolites were identified using an in-house library of standards. Among the known 77 metabolites, 52 metabolites (68%) were altered at p < 0.05 and 43 metabolites (56%) were altered at q < 0.05 at 24 hrs post differentiation compared to undifferentiated 3T3-L1 cells. Among the 16,922 detected features, 5,991 features (35%) were altered at p < 0.05 and 4,637 features (27%) were altered at q < 0.05 at 24 hrs (S1 Table).

To validate our time course metabolomics approach for the analysis of differentiating 3T3-L1 preadipocytes, we targeted several metabolites post data acquisition that have previously been reported to change during adipocyte differentiation and are thought to be important for regulation of the differentiation process.

**Fig 2:**
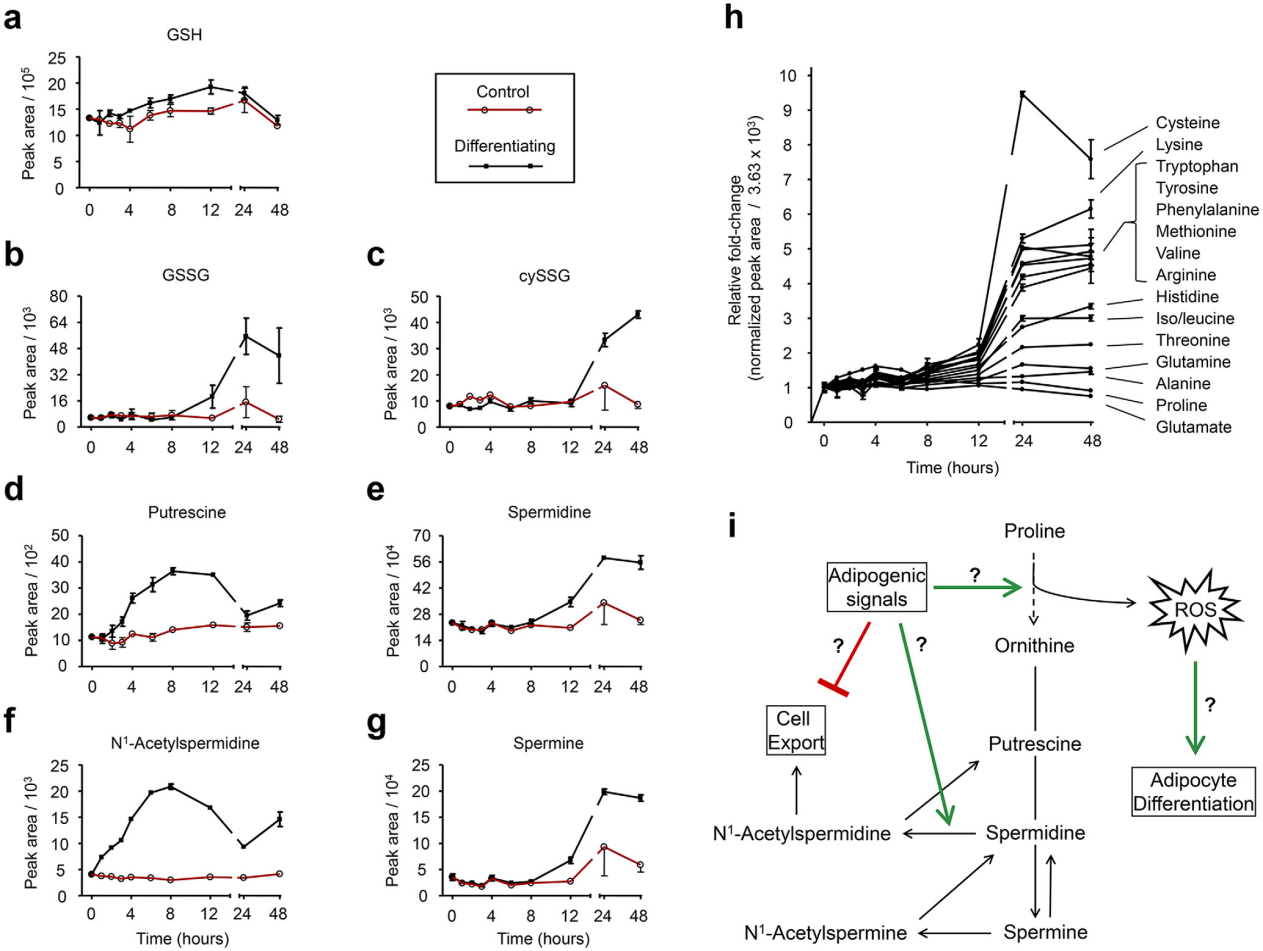
Temporal changes in polyamine, glutathione, and amino acid metabolism during 3T3-L1 preadipocyte differentiation. Time course profiles of metabolites involved in glutathione metabolism (A-C), polyamine metabolism (D-G), and amino acid metabolism (H) over the first 48 h of adipocyte differentiation. Control cells had 0.1% DMSO. (I) Hypothetical scheme linking proline degradation to reactive oxygen species (ROS) formation and polyamine biosynthesis in differentiating 3T3-L1 preadipocytes. n = 3 biological replicates for all time points except time 0 h, where n = 6. Shown are means ± SE.

Glutathione is the major endogenous, cellular antioxidant, and altering the ratio of reduced (GSH) to oxidized (GSSG) glutathione can affect protein glutathionylation status, potentially having a great impact on cell function. Previous studies have suggested an important role for glutathione during adipocyte differentiation. Within the first 24 h, the GSH/GSSG ratio decreases, shifting the cell towards a more oxidized state [22]. Exogenous glutathione or a precursor N-acetylcysteine (NAC) dampen adipogenesis, while glutathione depletion or exogenous oxidants enhance adipogenesis [22, 30]. Results from our time-course metabolomics experiment agree with the changes previously observed in glutathione metabolism. We found a substantial increase in GSSG at 24 and 48 h accompanied by little change in GSH (Fig 2a and 2b). We also detected an increase in cysteineglutathione disulfide (cySSG), an oxidation product of cysteine and GSH (Fig 2c).

Polyamines are small, aliphatic polycations essential for the life of virtually all organisms. The major polyamines are putrescine, spermidine, and spermine, and they are synthesized in that respective order from ornithine. Polyamines play crucial roles in cell growth and differentiation and have been found necessary for the differentiation of 3T3-L1 preadipocytes into mature adipocytes [16, 24–26]. In agreement with these previous studies, we found an increase in the polyamines spermine and spermidine during differentiation and these two molecules had very similar time course profiles (Fig 2d and 2e). Interestingly, the time course profile of putrescine matched that of *N*^1^-acetylspermidine, where we observed an increase up to 8 h post-differentiation, followed by a significant normalization by 24 h (Fig 2f and 2g). Under normal conditions, the formation of *N*^1^-acetylspermidine by spermidine/spermine *N*^1^-acetyltransferase (SSAT) is considered the limiting step in the removal of intracellular polyamines [31]. Because *N*^1^-acetylspermidine is rapidly exported from the cell, intracellular concentrations of this polyamine typically remain low. Our observation that *N*^1^-acetylspermidine was increased 7-fold 8 h after initiating differentiation, and that its oxidation product, putrescine, had a similar time course profile, suggests that polyamine export may be inhibited and perhaps an initial regulatory event of polyamine metabolism during adipocyte differentiation. However, it has previously been reported that SSAT activity increases during adipocyte differentiation [16] and separately, that over-expression of SSAT is accompanied by an increase in polyamine biosynthesis to support the increased export [32]. Therefore, our results may also be indicative of enhanced SSAT activity and polyamine export accompanied by compensatory polyamine biosynthesis.

In this study, we were able to detect and identify 15 amino acids (Fig 2h). Amino acids were mostly increased 24 and 48 h post differentiation with the exception of proline and glutamate, which were decreased at 48 h compared to earlier time points (Fig 2h). The relative changes in amino acids during adipocyte differentiation were found to be very consistent, as demonstrated by plotting the fold-changes for each amino acid from two separate experiments (S1 Fig). From this plot, it is clear that proline was consistently decreased the greatest 24 h after differentiation. Interestingly, proline is a precursor to ornithine, the immediate precursor for the biosynthesis of polyamines (Fig 2i). In addition, proline oxidation by the mitochondrial enzyme proline dehydrogenase can produce reactive oxygen species (ROS) [33], which were recently suggested to serve a regulatory role in a model of lifespan extension in *C*. *elegans* [34]. And several studies now describe a role for ROS as promoters of adipogenesis, specifically by accelerating mitotic clonal expansion. The decrease in proline observed here, especially relative to the increase in most other amino acids, may reflect enhanced degradation to not only supply precursors for polyamine biosynthesis, but perhaps to also produce pro-adipogenic ROS, in addition to the ROS from complex III [35] and NADPH oxidase [36]. In fact, it has previously been observed that the transcription factor PPARγ, a driving and necessary force of adipocyte differentiation, induces the expression of proline oxidase [37], and it has been proposed that proline oxidase is an important mediator of PPARγ-stimulated ROS production [38].

### Short-chain peptides increase during 3T3-L1 preadipocyte differentiation

**Fig 3:**
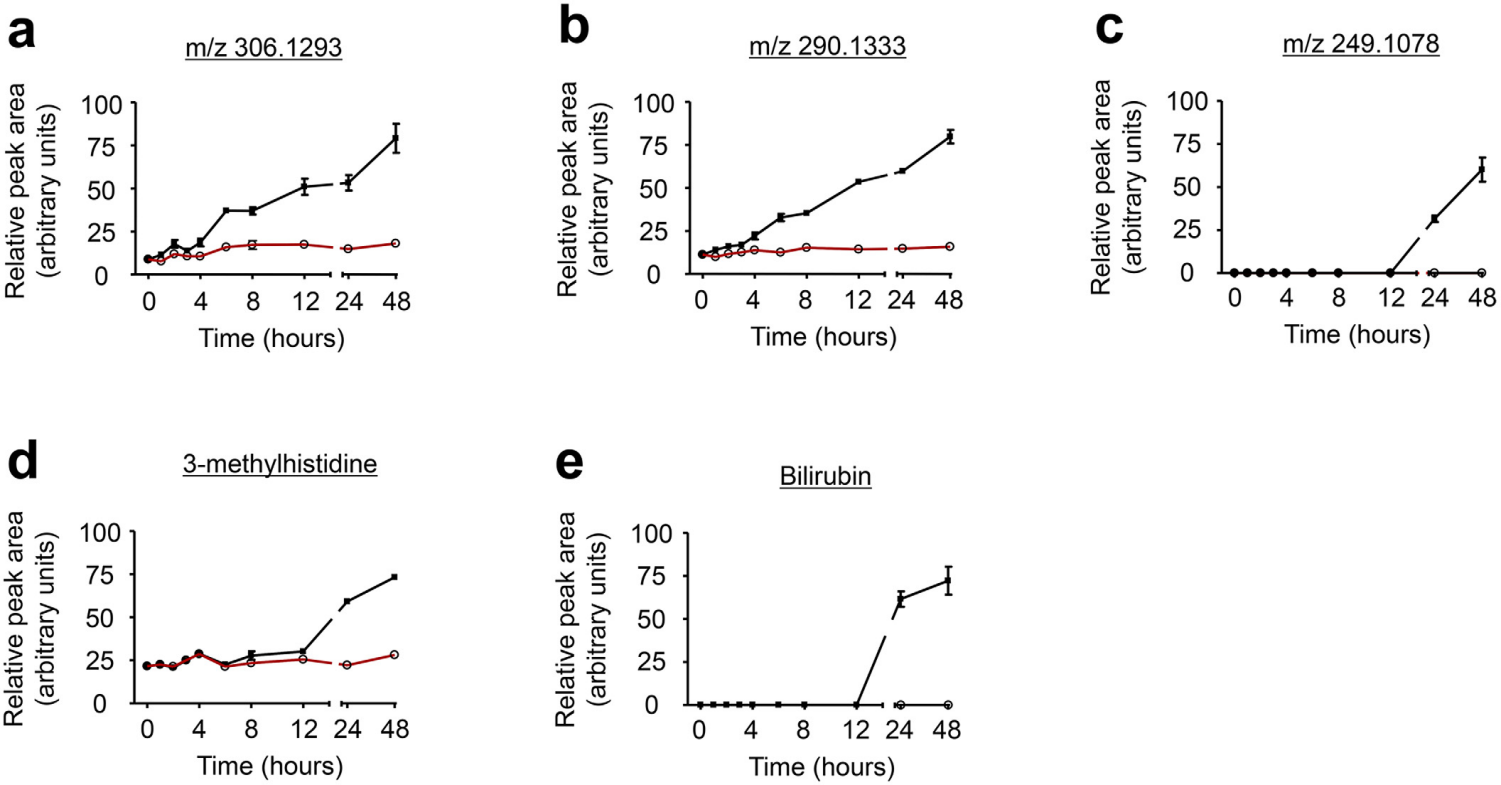
Temporal changes in products of protein degradation during adipocyte differentiation. Time course profiles of short-chain peptides (A-C), methylhistidine (D), and bilirubin (E) over the first 48 h of adipocyte differentiation. Control cells had only 0.1% DMSO. n = 3 biological replicates for all time points except time 0 h, where n = 6. Shown are means ± SE.

Using an untargeted metabolomics approach, we uncovered an increase in the formation of three short-chain peptides during adipocyte differentiation (Fig 3a–3c). We initially identified these peptides by accurate mass measurements (by the M+H ion) using the METLIN online metabolite database. The tripeptide that showed a molecular ion [M+H]^+^ at m/z 306.1293 produced prominent fragment ions with m/z 177.0853 (*y*_2_ ion), m/z 159.0741 (*y*_2_–H_2_O), and m/z 74 (*y*_1_–HCOOH), consistent with the sequence Glu-Gly-Thr. A second peptide (m/z 249.1062 [M+H]^+^) produced fragment ions with m/z 231.0969 [MH–H_2_O]^+^, m/z 120.0645 (*y* ion), m/z 102.0557 (*y*–H_2_O), m/z 84.0447 (*y*–2H_2_O), m/z 74 (*y*–HCOOH), and m/z 130.0488 (*b* ion), consistent with the sequence Glu-Thr. A third metabolite, m/z 290.1333, retrieved several matches in METLIN within 5 ppm and all were tripeptides containing the amino acids glutamate, alanine, and alanine, or glycine, valine, and aspartate, or serine, serine, and proline. The tripeptide’s sequence could not be established due to low abundance of its fragment ions.

Short-chain peptides are ubiquitous products of protein degradation and in some cases, products of biosynthesis. The peptides described in this study were increased 2 to 4-fold during the first 24 h of adipocyte differentiation (Fig 3a–3c), a change not explainable by the mild increase (30%) in total protein content (S2 Fig). These changes are suggestive of enhanced protein degradation during adipocyte differentiation. 3-Methylhistidine, a post-translationally modified amino acid and product of protein degradation that is not reutilized for protein synthesis [39], was also increased 24 and 48 h after initiating adipocyte differentiation, supporting the notion of up-regulated protein degradation during this period (Fig 3d). Also in support of enhanced protein degradation is the observation that bilirubin, a degradation product of heme-containing proteins [40], was undetectable in control, undifferentiating 3T3-L1 preadipocytes, but consistently present in differentiating adipocytes (Fig 3e). Together these results strongly suggest an increase in protein degradation during the early stages of 3T3-L1 preadipocyte differentiation.

### Short-chain peptides are products of peptide hydrolysis

**Fig 4:**
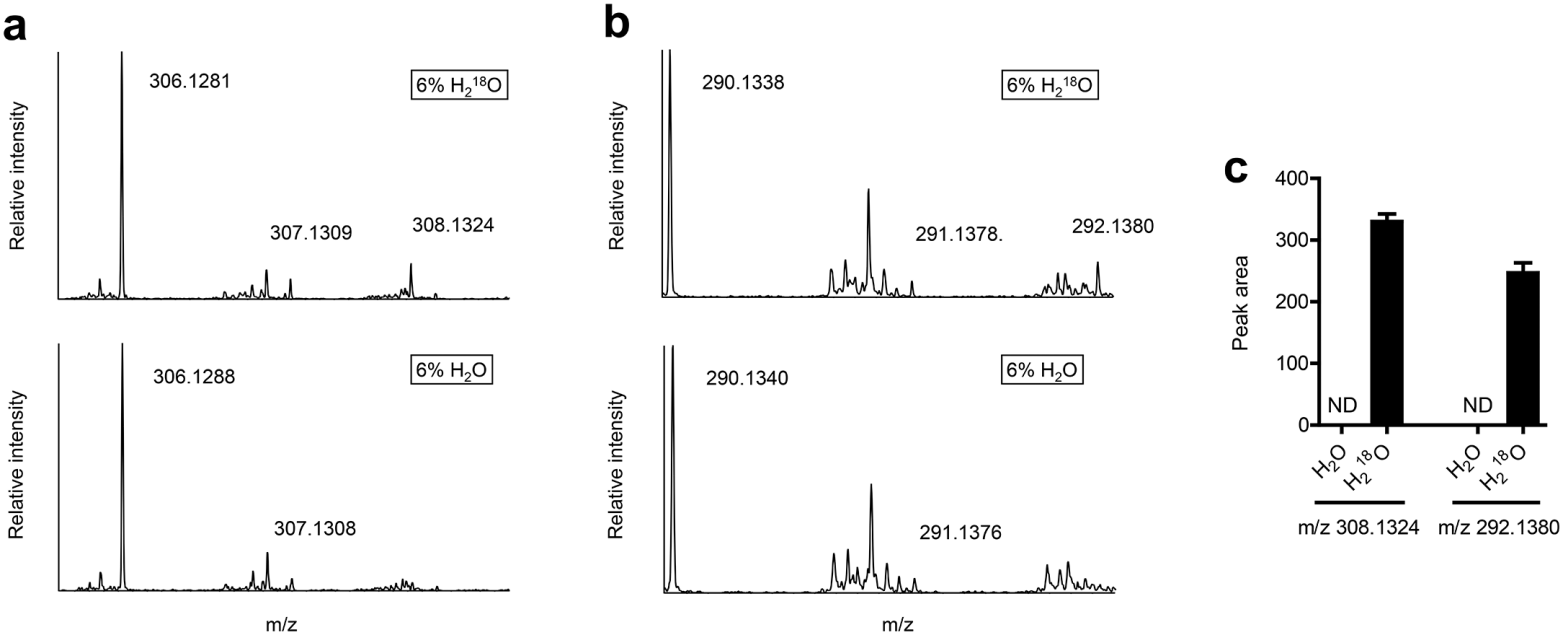
Short-chain peptides are products of peptide hydrolysis. Representative total ion chromatograms (A, B) and peak areas of peptide M+H+2 peaks (C) of differentiating (for 24 h) 3T3-L1 preadipocytes treated with 6% H_2_O or H_2_^18^O. Shown are means ± SE, n = 3 biological replicates. ND, not detected.

To confirm that the short-chain peptides described in this study are products of peptide or protein hydrolysis, we incubated differentiating 3T3-L1 preadipocytes with ^18^O labeled water (H_2_^18^O) and as a control, unlabeled water (H_2_O). As expected, ^18^O was consistently incorporated into the short-chain peptides (Fig 4), confirming them at least partially as products of hydrolysis and potential markers of protein and/or peptide degradation.

### Metabolomics of H_2_^18^O-treated differentiating 3T3-L1 preadipocytes reveals CDP-choline cycle activity and glutaminolysis

**Fig 5:**
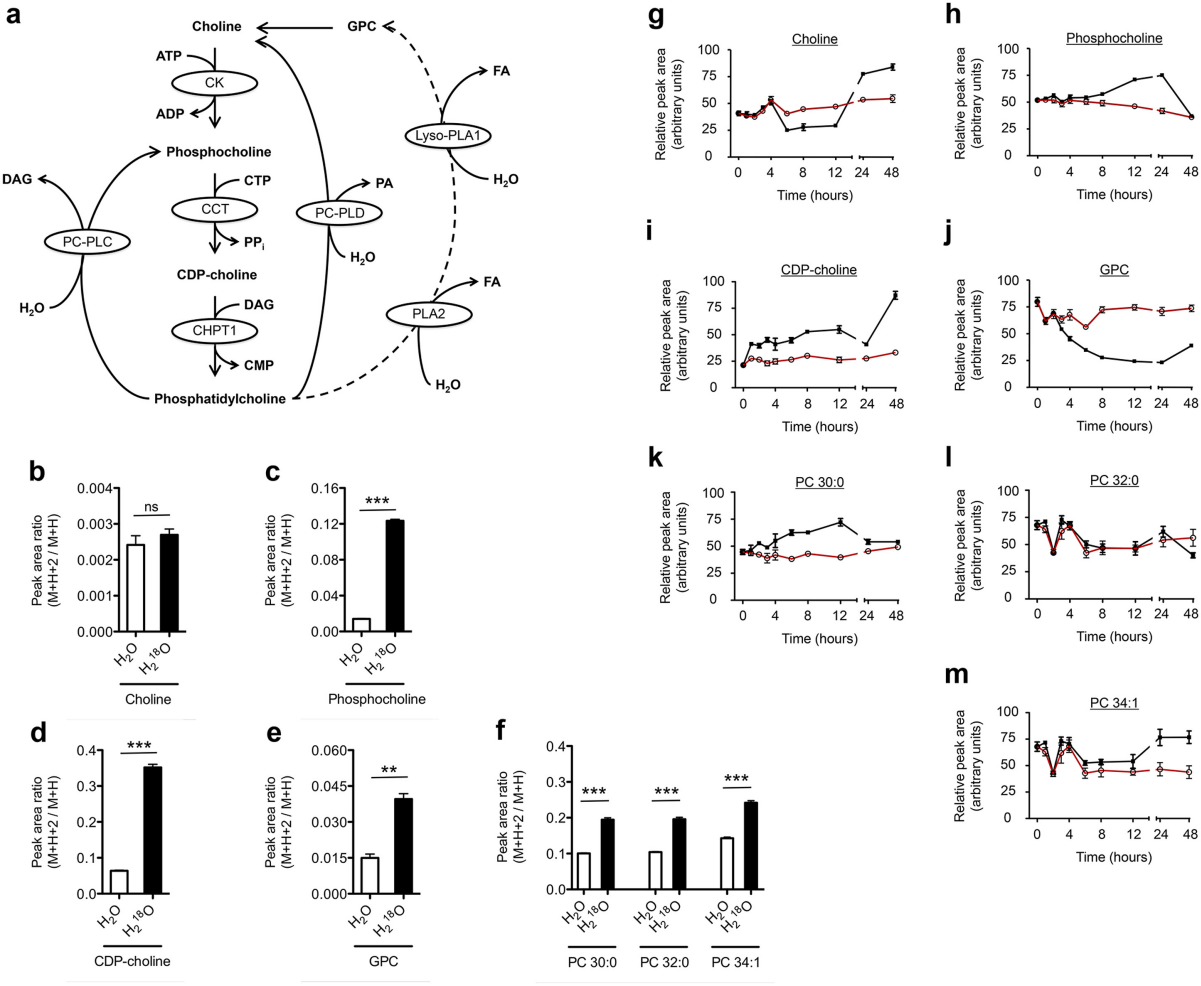
The CDP-choline cycle is active during 3T3-L1 preadipocyte differentiation. (A) Choline metabolism and the potential for ^18^O incorporation from H_2_^18^O. Adapted from Fagone and Jackowski [41]. (B-F) Peak area ratios (M+H+2 peak over the M+H peak, a measure of ^18^O incorporation) of choline metabolites. (G-M) Temporal changes in choline metabolites during 3T3-L1 preadipocyte differentiation. Shown are means ± SE, n = 3 biological replicates except time 0 h, where n = 6. *** p < 0.0001 and ** p < 0.001 from a two-tailed t-test. ns, not significant where p > 0.05.

An untargeted metabolomic analysis of differentiating adipocytes treated with H_2_^18^O revealed incorporation of ^18^O into several choline metabolites, including phosphocholine, CDP-choline, glycerophosphocholine (GPC), and several phosphatidylcholine (PC) species (Fig 5). PC is a major component of biological membranes and is considered an intermediate of choline metabolism, rather than an endpoint [41]. The synthesis and degradation of PCs is a highly regulated process that is linked to cell cycle and is essential in proliferating cells [42, 43]. When quiescent cells are stimulated to enter the G1 phase, the activity of the flux-regulating enzyme of PC synthesis, phosphocholine cytidyltransferase, increases [42]. The stimulation of PC synthesis is balanced by PC degradation in the G1 phase and is mediated by the hydrolytic enzymes phospholipase A and neuropathy target esterase [44, 45]. Growth arrested, post-confluent 3T3-L1 preadipocytes undergo about two rounds of cell division after initiating differentiation, a process known as mitotic clonal expansion (MCE), which is required for the complete differentiation of 3T3-L1 cells into mature adipocytes [46]. Since MCE occurs within the first 48 h of initiating differentiation [46], alterations to choline metabolism would not be unexpected. Indeed, our results show incorporation of ^18^O into both anabolic (CDP-choline) and catabolic (GPC) components of choline metabolism (Fig 5), suggestive of CDP-choline cycle activity and cell membrane remodeling.

These results provide evidence that there is flux through the CDP-choline cycle early in the differentiation of 3T3-L1 preadipocytes into mature adipocytes.

**Fig 6:**
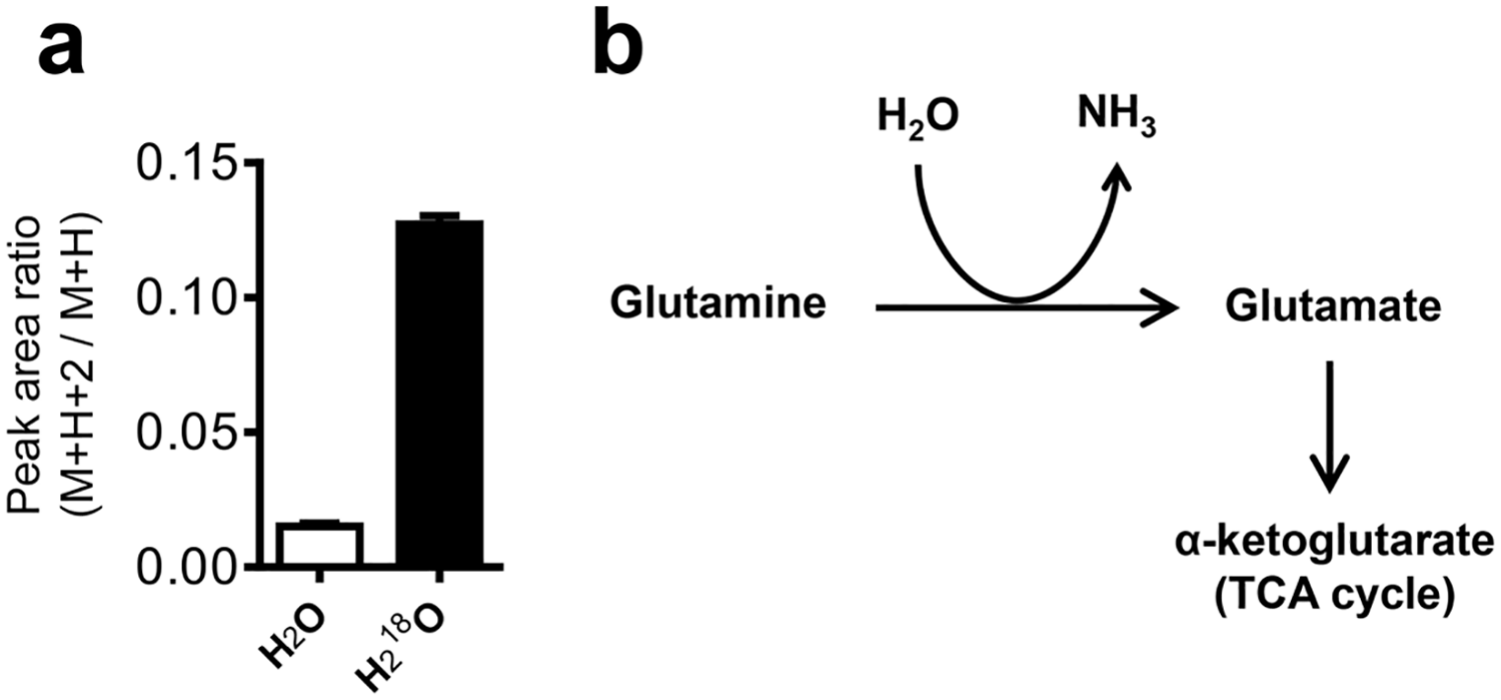
Glutaminolysis is active during 3T3-L1 preadipocyte differentiation. (A) Peak area ratio (M+H+2 peak over the M+H peak, a measure of ^18^O incorporation) of glutamate. (B) Scheme of glutamine hydrolysis for production of the anaplerotic amino acid glutamate. Shown are means ± SE, n = 3 biological replicates.

In addition to choline metabolites, our metabolomics analysis of differentiating 3T3-L1 preadipocytes incubated with H_2_^18^O revealed significant incorporation of ^18^O into glutamate, a product of glutamine hydrolysis and major anaplerotic amino acid (Fig 6). These results suggest that glutaminolysis is active in differentiating 3T3-L1 cells during MCE, despite the observed decrease in total glutamate with the unlabeled metabolomics experiment. The flux through glutamate is likely in part destined for entry into the TCA cycle to support the increased energy and biomass requirements associated with cell division. Indeed, both mitochondrial and glycolytic energy production are increased 24 h after initiating 3T3-L1 differentiation, as evidenced by the increase in the rates of cellular oxygen consumption and extracellular acidification (S3 Fig), a measure of lactate export. These results highlight the utility of metabolomics coupled with H_2_^18^O labeling to uncover changes in cellular metabolism that were otherwise undetectable by time course metabolomics.

### Short-chain peptide levels in differentiating 3T3-L1 preadipocytes are associated with proteasome and matrix metalloproteinase activity, but not autophagy

Protein degradation during adipocyte differentiation is a complex and highly regulated process. The ubiquitin-proteasome system is required for the degradation of anti-adipogenic proteins during adipocyte differentiation [47] and proteasome inhibition decreases adipocyte differentiation [48]. Autophagy also appears to be important for adipocyte differentiation. Inhibition of autophagy in adipocytes through adipose specific deletion of autophagy-related genes decreases white adipose tissue mass in animals [49, 50]. Though the ubiquitin-proteasome and lysosome pathways are the major sources of protein degradation and short-chain peptide formation, matrix metalloproteinases, which aid in the remodeling of the extracellular matrix, also regulate adipocyte differentiation, as their inhibition prevents adipocyte differentiation and many are up-regulated with obesity [51, 52].

**Fig 7:**
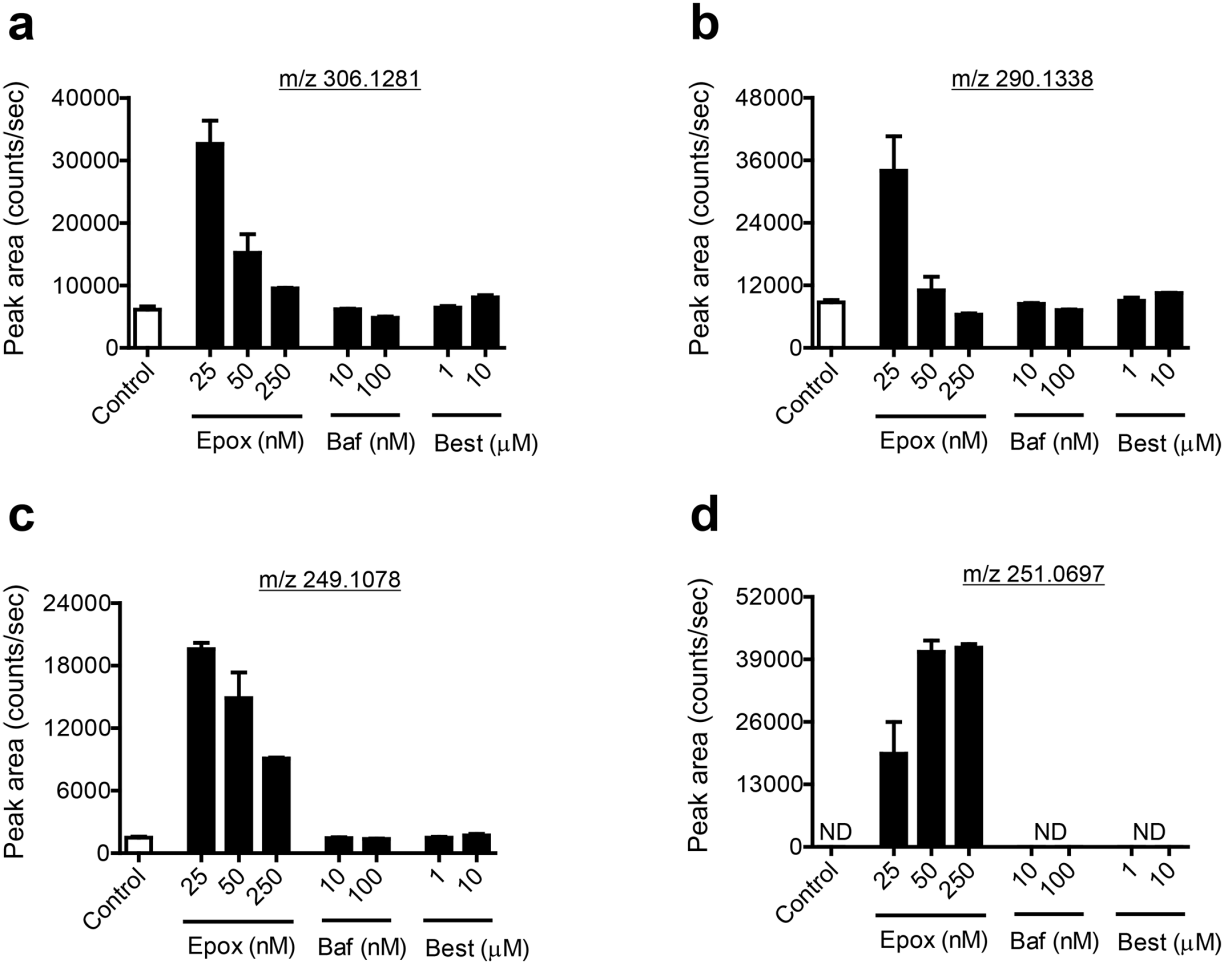
Short-chain peptide levels are reflective of proteasome activity. (A-D) Relative levels of short-chain peptides in the presence of various concentrations of inhibitors of proteasome activity (epoxomicin; Epox), autophagosome-lysosome fusion (bafilomycin A1; Baf), and aminopeptidase activity (bestatin; Best). 0.1% DMSO served as a vehicle control. Shown are means ± SE, n = 3 biological replicates. ND, not detected.

To determine whether the short-chain peptides detected in this study are products of autophagy, we treated differentiating adipocytes with bafilomycin A1, an inhibitor of autophagosome-lysosome fusion. To determine if the proteasome played a role in the formation of these short-chain peptides, we treated differentiating adipocytes with the selective proteasome inhibitor, epoxomicin. To determine if matrix metalloproteinases were responsible for the formation of the peptides, we treated differentiating adipocytes with batimastat, a broad-spectrum inhibitor of matrix metalloproteinases. We also treated cells with bestatin, an aminopeptidase inhibitor, to determine if the short-chain peptide levels are affected by aminopeptidase activity. Bafilomycin A1 and bestatin had no effect on the levels of the short-chain peptides described in this study. Epoxomicin treatment unexpectedly resulted in an increase of all peptides (3.9 to 13.1-fold) which was most significant at the lowest dose level, 25 nM (Fig 7). An additional peptide, with an m/z value of 251.0697 in positive ion mode eluting at 5.7 minutes, was detected only in the presence of epoxomicin (Fig 7). A search for this m/z value in METLIN yielded only three results within 5 ppm and all were peptides containing the amino acids cysteine and glutamate. It produced fragment ions with m/z 122.0274 (*y*_1_ ion), m/z 84.0446 (*a*_1_–H_2_O), and m/z 234.0435 [MH–NH_3_]^+^ on collisional activation, consistent with the sequence Glu-Cys or 03B3Glu-Cys.

**Fig 8:**
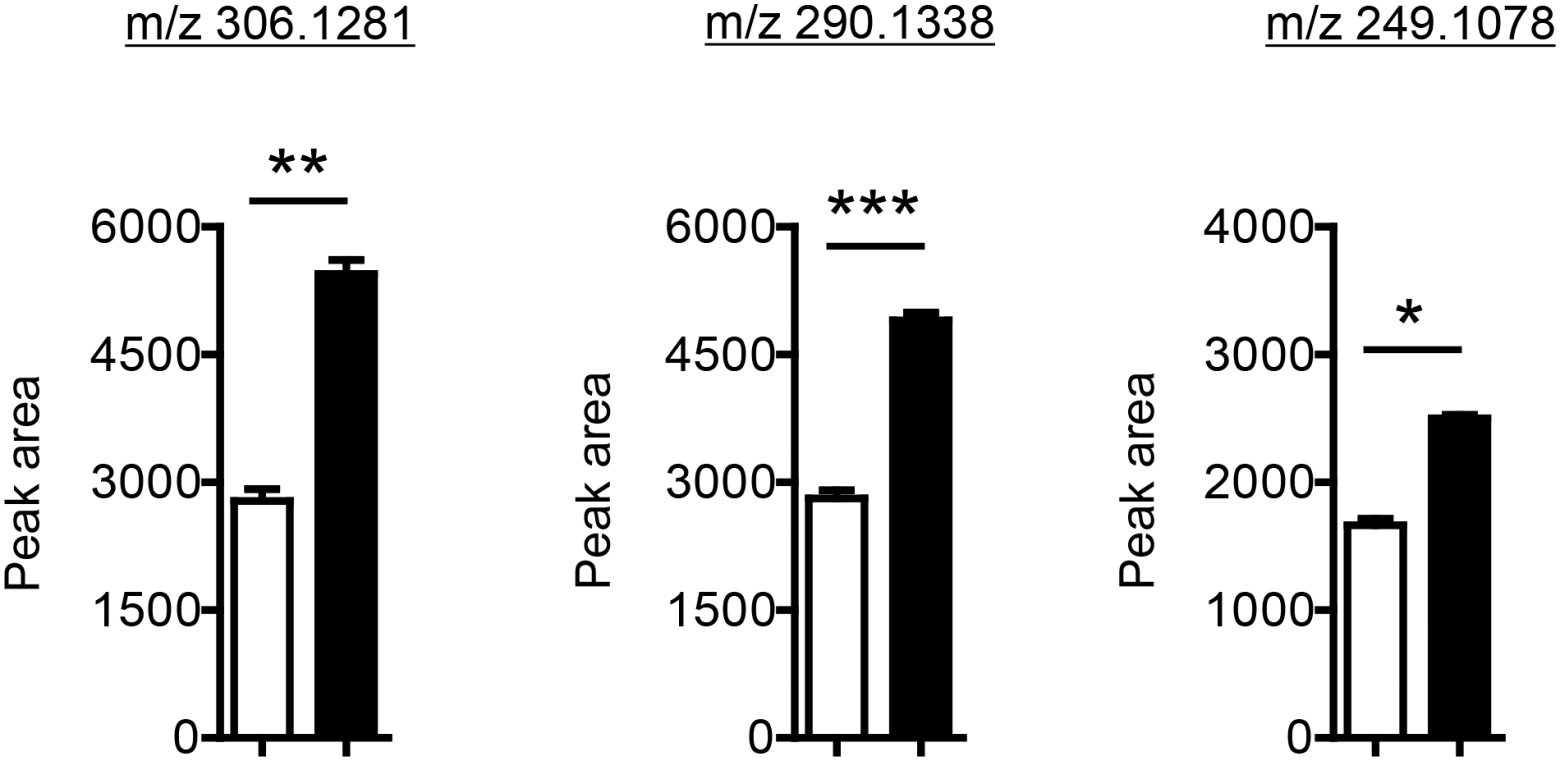
Short-chain peptide levels are reflective of matrix metalloproteinase activity. (A-C) Relative levels of short chain peptides in the presence of batimastat (10 μM), a broad spectrum matrix metalloproteinase inhibitor. 0.1% DMSO served as a vehicle control. Shown are means ± SE, n = 3 biological replicates. *** p < 0.0001 and ** p < 0.001 and * p > 0.05 from a two-tailed t-test.

Treating differentiating adipocytes with batimastat had an effect similar to that of epoxomicin, where all peptides increased, though the increase was smaller (1.6 to 2.0-fold) (Fig 8). Though it is unclear why these peptides increased in the presence of inhibitors of protein degradation instead of decreasing as would be expected if the peptides were products of protein degradation, a recent peptidomic analysis of human embryonic kidney cells treated with epoxomicin describes a similar phenomenon, where a small subset of peptides increased in the presence of epoxomicin, presumably due to a specific increase of the activity of the beta-1 subunit of the proteasome [53].

### Short-chain peptide formation is not driven by ROS in differentiating 3T3-L1 preadipocytes

**Fig 9:**
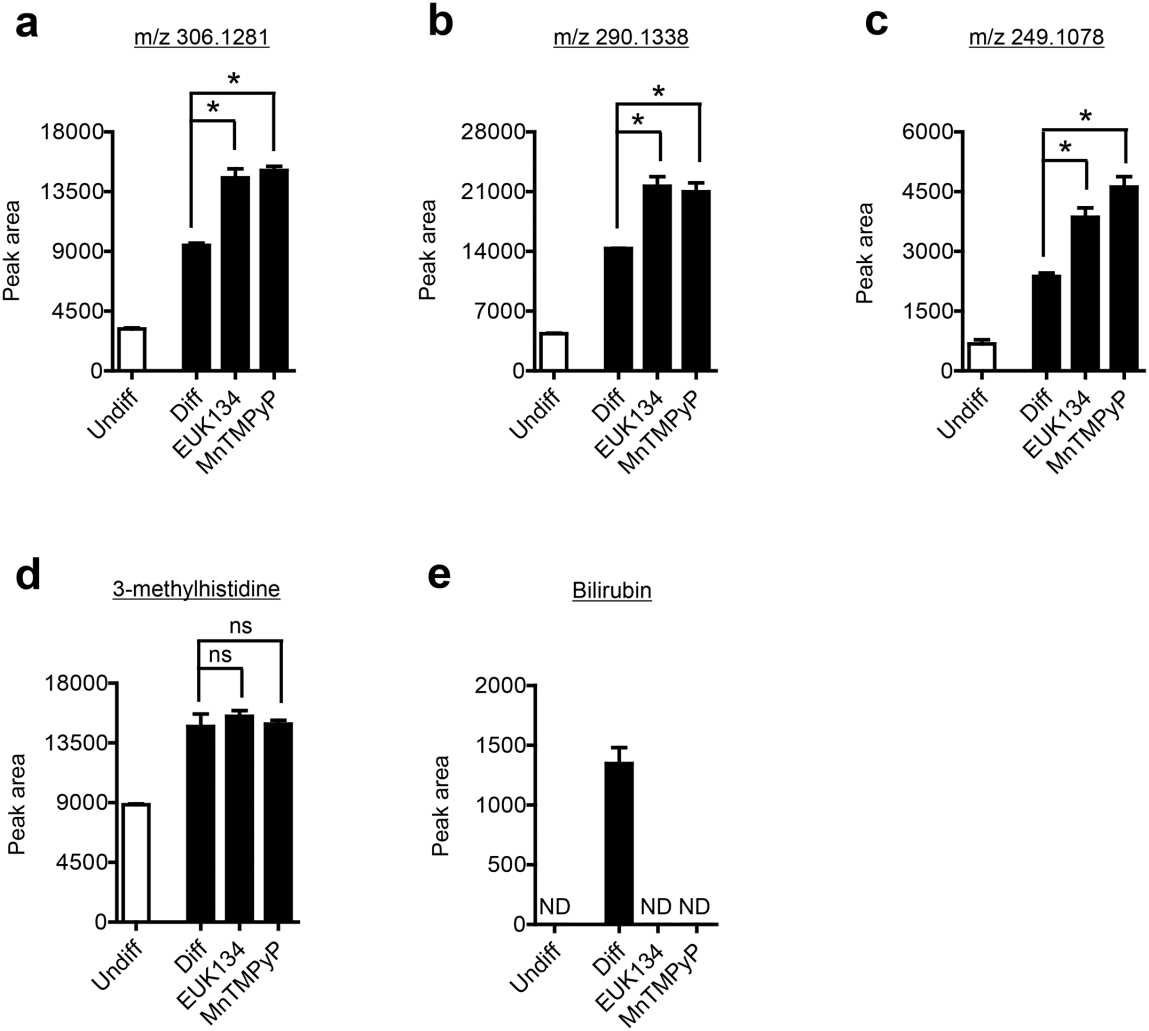
Metalloporphyrin complex antioxidants alter the levels of short-chain peptides and bilirubin. Relative levels of short chain peptides (A-C), methylhistidine (D), and bilirubin (E) in control undifferentiating 3T3-L1 preadipocytes (Undiff) and differentiating 3T3-L1 readipocytes (Diff) in the presence or not of the metalloporphyrin complex antioxidants EUK134 or nTMPyP. Plain media served as a vehicle control. Shown are means ± SE, n = 3 biological replicates. * p < 0.05 from a Tukey post-hoc test following a one-way ANOVA. ND, not detected.

Many reports have described a role for ROS in the regulation of protein degradation. In fact, ROS are thought to play a role in the regulation of proteasomal [54] and autophagosomal [55] protein degradation as well as matrix metalloproteinase activity [56]. In addition, ROS from mitochondrial complex III and NADPH oxidase are increased during adipocyte differentiation and are thought to serve a pro-adipogenic regulatory role, since antioxidants diminish lipid accumulation in differentiating adipocytes [23, 35, 36]. These previous observations prompted us to explore the role of ROS in the increased formation of short-chain peptides during adipocyte differentiation. To determine whether ROS were responsible for the formation of the short-chain peptides, we differentiated adipocytes in the presence of the metalloporphyrin complexes MnTMPyP and EUK134, both of which serve as cell membrane-permeable superoxide and peroxynitrite dismutase/catalase mimetics. Unexpectedly, all three peptides were slightly increased (1.5 to 2.0-fold) in the presence of MnTMPyP or EUK134 (Fig 9a–9c). However, MnTMPyP and EUK134 had no effect on the level of 3-methylhistidine (Fig 9d). Interestingly, both MnTMPyP and EUK134 completely prevented the formation of bilirubin during adipocyte differentiation (Fig 9e), suggesting ROS may be responsible for bilirubin production. However, it is well known that many metalloporphyrin complexes, given their structural similarities to heme, can act as inhibitors of heme oxygenase 1 [57], the enzyme that converts heme to the bilirubin precursor, biliverdin.

Based on these previous reports, we hypothesize that the prevention in the formation of bilirubin by MnTMPyP and EUK134 in differentiating adipocytes is the result of direct inhibition of heme oxygenase 1 activity, and not a ROS mediated mechanism. Metalloporphyrin inhibitors of heme oxygenase 1 possess other off-target effects including inhibition of nitric oxide synthase and soluble guanylyl cyclase [58], suggesting caution should be used when interpreting data from experiments where only MnTMPyP and EUK134 are utilized. In fact, treating differentiating adipocytes with PEG-catalase or hydrogen peroxide had little effect on the levels of the three peptides, methylhistidine, or bilirubin (S4 Fig), suggesting the increase in the peptides by the metalloporphyrin complexes may be an off target effect, along with the inhibited bilirubin formation.

The glutathione precursor NAC has been used with success to inhibit adipogenesis. However, NAC was not used in this study since we found that relatively low concentrations of NAC dose-dependently depleted short-chain peptides in 3T3-L1 preadipocytes after a 12 h incubation (S5 Fig). Because NAC has been reported to inhibit 26S proteasome activity [59], we propose that NAC depleted the peptides described in this study through proteasome inhibition and this may offer an alternative or additional mechanism for how NAC reduces adipocyte differentiation, since proteasome inhibition appears sufficient to decrease the differentiation of adipocytes [48].

## Conclusions

Using an untargeted metabolomics approach, we uncovered an increase in the levels of several intracellular di- and tripeptides during the early phase of 3T3-L1 preadipocyte differentiation. When differentiating 3T3-L1 preadipocytes were treated with H_2_^18^O, ^18^O was incorporated into the short-chain peptides, and the levels of the peptides were altered by proteasome and matrix metalloproteinase inhibition, confirming these molecules as products, and potential markers, of peptide and/or protein hydrolysis. In addition, a metabolomics analysis of H_2_^18^O-treated, differentiating 3T3-L1 preadipocytes revealed flux through the CDP-choline cycle and glutaminolysis in differentiating 3T3-L1 preadipocytes, highlighting the potential for isotope labeling experiments to uncover active metabolic pathways during fundamental cellular processes and the inability of single point or time course metabolomics experiments to detect such changes. Though 3T3-L1 preadipocytes have been a robust model of adipocyte differentiation, metabolism in human adipocytes can differ and future efforts will be necessary to confirm these findings in human adipocytes.

## Supporting Information

S1 Fig The relative changes in amino acid levels during 3T3-L1 preadipocyte differentiation are consistent. Fold-changes (undifferentiating vs. 24 h differentiating 3T3-L1 preadipocytes) for all amino acids detected (except cysteine, which has been removed for clarity due to large fold-change) from two separate experiments plotted against each other.
(PDF)

S2 Fig Effect of 3T3-L1 preadipocyte differentiation on total cellular protein content. Change in total cellular protein content after 24 h of 3T3-L1 preadipocyte differentiation in the presence and absence of metalloporphyrin complex antioxidants. Plain media served as a vehicle control. Shown are means ± SE, n = 3. *** p < 0.001 from a Tukey post-hoc test following a one-way ANOVA. ns, not significant where p > 0.05; Ctrl, control; E, EUK134; M, MnTMPyP.
(PDF)

S3 Fig Effect of 3T3-L1 preadipocyte differentiation on OCR and ECAR. Changes in the oxygen consumption rate (OCR) (A) and extracellular acidification rate (ECAR) (B) after 24 h of 3T3-L1 preadipocyte differentiation in the presence and absence of metalloporphyrin complex antioxidants. Plain media served as a vehicle control. Shown are means ± SE, n = 5. *** p < 0.001 and * p < 0.05 from a Tukey post-hoc test following a one-way ANOVA. ns, not significant; Ctrl, control; E, EUK134; M, MnTMPyP.
(PDF)

S4 Fig Reactive oxygen species do not play a major role in short-chain peptide formation during 3T3-L1 preadipocyte differentiation. Relative levels of short chain peptides, 3-methylhistidine, and bilirubin in differentiating 3T3-L1 preadipocytes treated with various concentrations of H_2_O_2_ or PEG-catalase. Plain media served as a vehicle control. Shown are means ± SE, n = 3.
(PDF)

S5 Fig Effect of NAC on short-chain peptide levels in 3T3-L1 preadipocytes. Changes in short-chain peptide levels in 2-day post-confluent 3T3-L1 preadipocytes after a 12 h incubation with various concentrations of *N*-acetylcysteine (NAC). Plain media served as a vehicle control. Shown are means ± SE, n = 3. ND, not detected.
(PDF)

S1 Table Identified metabolites. (XLSX)
