# Supplementary material for: 18O-Tracer Metabolomics Reveals Protein Turnover and CDP-Choline Cycle Activity in Differentiating 3T3-L1 Pre-Adipocytes
Source: PLoS One. 2016 Jun 8;11(6):e0157118. doi: 10.1371/journal.pone.0157118 (PMC4898700; doi:10.1371/journal.pone.0157118)

S1 Fig. The relative changes in amino acid levels during 3T3-L1 preadipocyte differentiation are consistent.

Fold-changes (undifferentiating vs. 24 h differentiating 3T3-L1 preadipocytes) for all amino acids detected (except cysteine, which has been removed for clarity due to large fold-change) from two separate experiments plotted against each other.

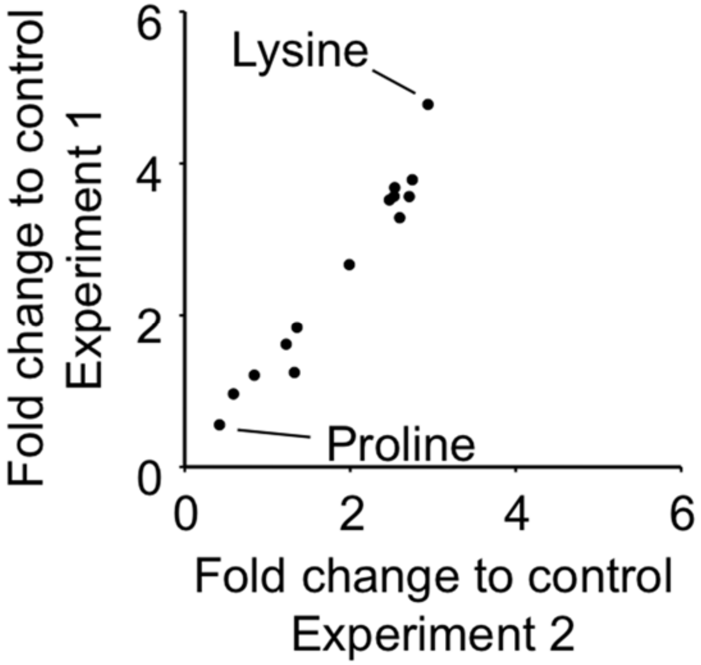

Supplement: S1 Fig — Fold-changes (undifferentiating vs. 24 h differentiating 3T3-L1 preadipocytes) for all amino acids detected (except cysteine, which has been removed for clarity due to large fold-change) from two separate experiments plotted against each other. (PDF) [file pone.0157118.s001.pdf]
