# Supplementary material for: 18O-Tracer Metabolomics Reveals Protein Turnover and CDP-Choline Cycle Activity in Differentiating 3T3-L1 Pre-Adipocytes
Source: PLoS One. 2016 Jun 8;11(6):e0157118. doi: 10.1371/journal.pone.0157118 (PMC4898700; doi:10.1371/journal.pone.0157118)

S2 Fig. Effect of 3T3-L1 preadipocyte differentiation on total cellular protein content.

Change in total cellular protein content after 24 h of 3T3-L1 preadipocyte differentiation in the presence and absence of metalloporphyrin complex antioxidants. Plain media served as a vehicle control.
Shown are means ± SE, n=3. *** $p < 0.001$ from a Tukey post-hoc test following a one-way ANOVA. ns, not significant where $p > 0.05$; Ctrl, control; E, EUK134; M, MnTMPyP.

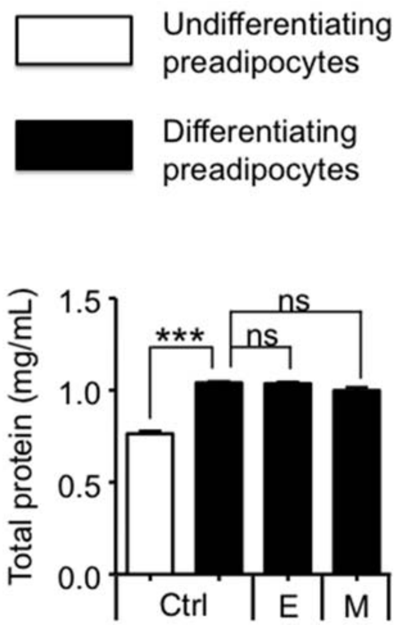

Supplement: S2 Fig — Change in total cellular protein content after 24 h of 3T3-L1 preadipocyte differentiation in the presence and absence of metalloporphyrin complex antioxidants. Plain media served as a vehicle control. Shown are means ± SE, n = 3. *** p < 0.001 from a Tukey post-hoc test following a one-way ANOVA. ns, not significant where p > 0.05; Ctrl, control; E, EUK134; M, MnTMPyP. (PDF) [file pone.0157118.s002.pdf]
