# Supplementary material for: 18O-Tracer Metabolomics Reveals Protein Turnover and CDP-Choline Cycle Activity in Differentiating 3T3-L1 Pre-Adipocytes
Source: PLoS One. 2016 Jun 8;11(6):e0157118. doi: 10.1371/journal.pone.0157118 (PMC4898700; doi:10.1371/journal.pone.0157118)

S3 Fig. Effect of 3T3-L1 preadipocyte differentiation on OCR and ECAR.

Changes in the oxygen consumption rate (OCR) (A) and extracellular acidification rate (ECAR) (B) after 24 h of 3T3-L1 preadipocyte differentiation in the presence and absence of metalloporphyrin complex antioxidants. Plain media served as a vehicle control.
Shown are means ± SE, n=5. *** $p < 0.001$ and * $p < 0.05$ from a Tukey post-hoc test following a one-way ANOVA. ns, not significant; Ctrl, control; E, EUK134; M, MnTMPyP.

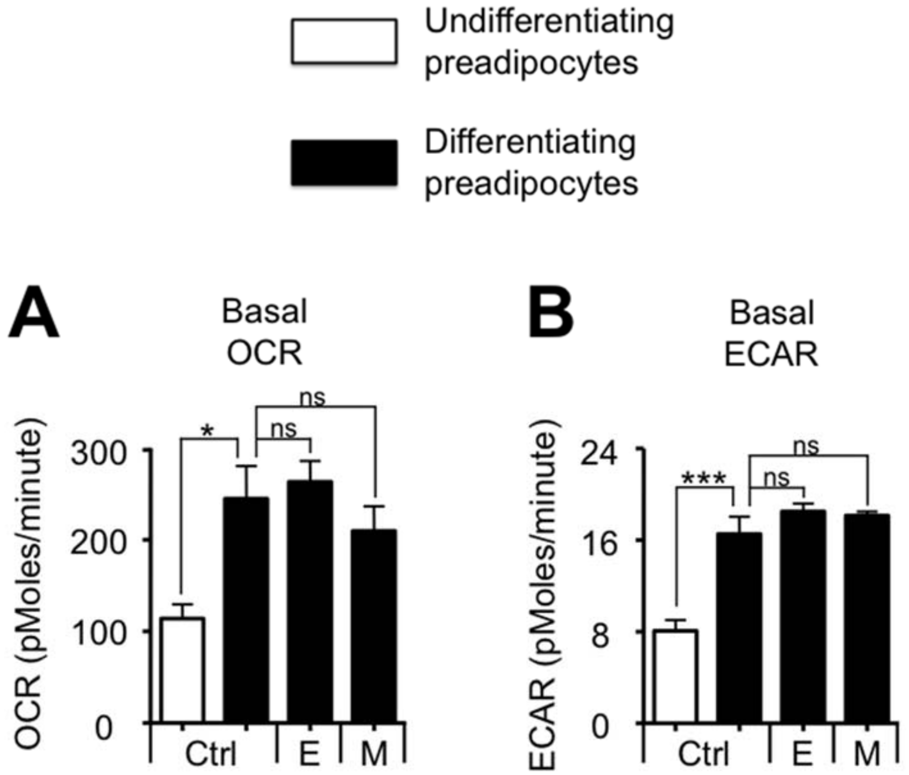

Supplement: S3 Fig — Changes in the oxygen consumption rate (OCR) (A) and extracellular acidification rate (ECAR) (B) after 24 h of 3T3-L1 preadipocyte differentiation in the presence and absence of metalloporphyrin complex antioxidants. Plain media served as a vehicle control. Shown are means ± SE, n = 5. *** p < 0.001 and * p < 0.05 from a Tukey post-hoc test following a one-way ANOVA. ns, not significant; Ctrl, control; E, EUK134; M, MnTMPyP. (PDF) [file pone.0157118.s003.pdf]
