# Supplementary material for: 18O-Tracer Metabolomics Reveals Protein Turnover and CDP-Choline Cycle Activity in Differentiating 3T3-L1 Pre-Adipocytes
Source: PLoS One. 2016 Jun 8;11(6):e0157118. doi: 10.1371/journal.pone.0157118 (PMC4898700; doi:10.1371/journal.pone.0157118)

S4 Fig. Reactive oxygen species do not play a major role in short-chain peptide formation during 3T3-L1 preadipocyte differentiation.

Relative levels of short chain peptides, 3-methylhistidine, and bilirubin in differentiating 3T3-L1 preadipocytes treated with various concentrations of H_2O_2 or PEG-catalase. Plain media served as a vehicle control.
Shown are means ± SE, n=3.

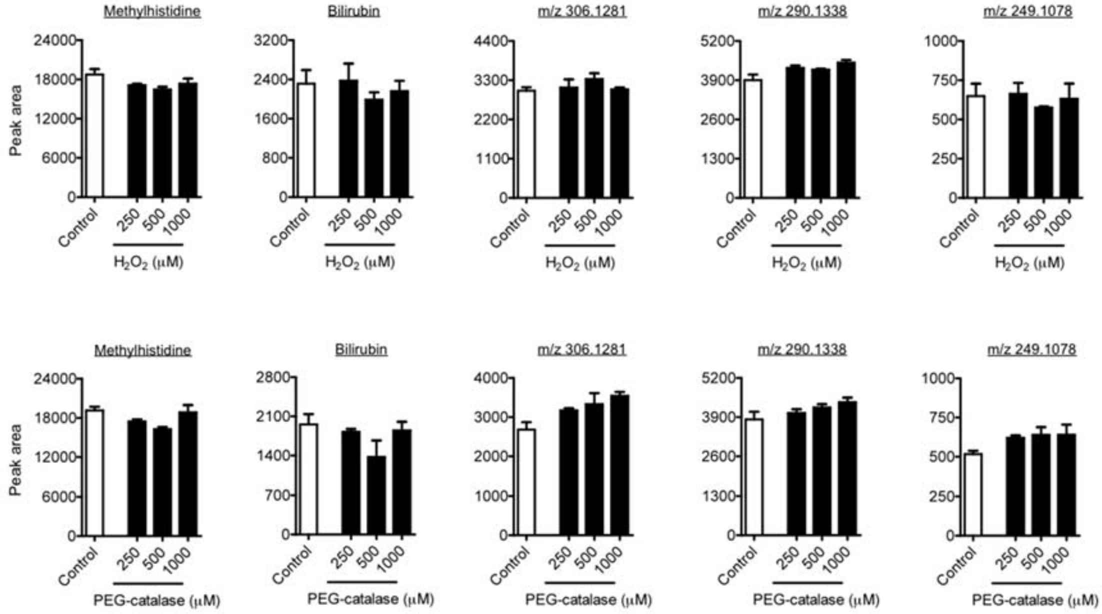

Supplement: S4 Fig — Relative levels of short chain peptides, 3-methylhistidine, and bilirubin in differentiating 3T3-L1 preadipocytes treated with various concentrations of H2O2 or PEG-catalase. Plain media served as a vehicle control. Shown are means ± SE, n = 3. (PDF) [file pone.0157118.s004.pdf]
