# Supplementary material for: 18O-Tracer Metabolomics Reveals Protein Turnover and CDP-Choline Cycle Activity in Differentiating 3T3-L1 Pre-Adipocytes
Source: PLoS One. 2016 Jun 8;11(6):e0157118. doi: 10.1371/journal.pone.0157118 (PMC4898700; doi:10.1371/journal.pone.0157118)

S5 Fig. Effect of NAC on short-chain peptide levels in 3T3-L1 preadipocytes.

Changes in short-chain peptide levels in 2-day post-confluent 3T3-L1 preadipocytes after a 12 h incubation with various concentrations of *N*-acetylcysteine (NAC). Plain media served as a vehicle control.
Shown are means ± SE, n=3. ND, not detected.

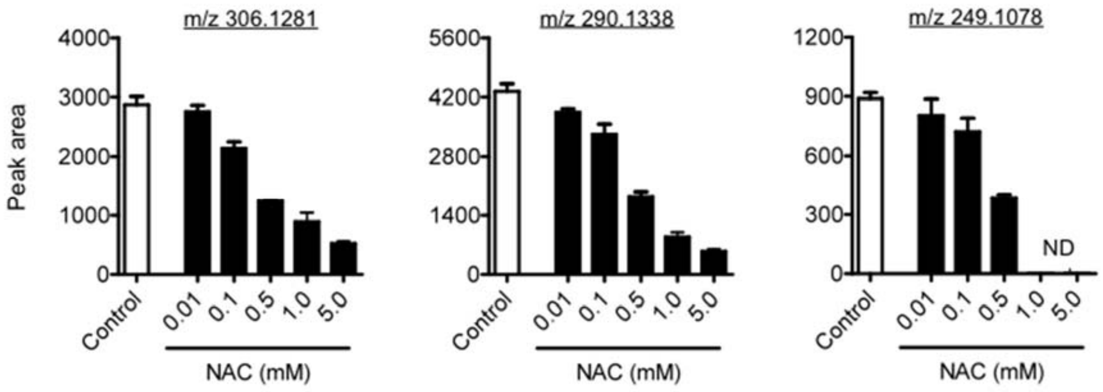

Supplement: S5 Fig — Changes in short-chain peptide levels in 2-day post-confluent 3T3-L1 preadipocytes after a 12 h incubation with various concentrations of N-acetylcysteine (NAC). Plain media served as a vehicle control. Shown are means ± SE, n = 3. ND, not detected. (PDF) [file pone.0157118.s005.pdf]
